# The Relationship Between Job Crafting and Nurses’ Happiness in Bisha Region, Kingdom of Saudi Arabia

**DOI:** 10.7759/cureus.35697

**Published:** 2023-03-02

**Authors:** Norah Alharthi, Naglaa Elseesy, Wafa Aljohani

**Affiliations:** 1 Nursing, King Abdulaziz University, Jeddah, SAU; 2 Public Health Nursing, Faculty of Nursing, King Abdulaziz University, Jeddah, SAU; 3 Nursing Administration, Faculty of Nursing, Alexandria University, Alexandria, EGY; 4 Medical-Surgical Nursing, Faculty of Nursing, King Abdulaziz University, Jeddah, SAU; 5 Nursing, Batterjee Medical College, Jeddah, SAU

**Keywords:** work happiness, relationship, nurses, job happiness, job crafting

## Abstract

Background

Job crafting is an example of constructive behavior in which workers aggregate resources to meet their needs and succeed at work. Individuals may change job boundaries and social relationships at their convenience to feel closer to what they consider the perfect workplace.

Aim

To analyze the relationship of job crafting with nurses' happiness.

Method

A quantitative cross-sectional study was conducted on 441 nurses from Saudi Arabia. Data were collected using an electronic questionnaire (Google Drive). This questionnaire includes demographic factors, a Job Crafting Scale (JCS), and the Oxford Happiness Questionnaire (OHQ). Ethical considerations were strictly followed in the present study.

Result

The results revealed that most nurses had a high level of job crafting. The overall mean score of JCS was (91.2 ± 11.8). The present results demonstrate that the overall mean happiness score was at a moderate level. The overall mean OHQ score was 3.98 ± 4.25, and there was a significant positive correlation between the OHQ score according to the increasing structural domain (r=0.246), decreasing hindering job demands (r=0.220), increasing social job resources (r=0.176), increasing challenging job demands (r=0.212), and the overall total JCS (r=0.252). This indicates that the increase in job happiness is correlated with the increase in job crafting.

Conclusion

Job crafting has a positive significant relation with nurses' happiness. It is the responsibility of nurse managers and educators in the healthcare industry to provide a suitable work environment, beginning with including employees in decision-making and duties through leadership empowerment and providing support programs, and activities to increase the job happiness and job crafting experienced by nurses.

## Introduction

Nurses experience high levels of stress while providing daily care to patients. To overcome this issue, it is important to proactively implement administrative regulations that facilitate a healthy and comfortable working environment and stimulate job crafting [1]. Job crafting enables nurses to create occupation models by altering the physical, cognitive, and relational components of the working activity [2].

Lichtenthaler and Fischbach defined job crafting as employee-driven modifications in concrete work role bounds and intangible work role perceptions [3]. Through this process, employees can enhance their work identities by recognizing the meaning of their work, growing and shrinking various relational aspects of their jobs, and modifying (i.e., increasing or decreasing) the number, scope, or nature of job tasks performed [4]. Romeo et al. noted that initiating changes in duties and interactions or even adjusting beliefs about the work is an example of job crafting, the active behavior that gives workers the opportunity to redesign their work environment [4,5].

Job crafting is classified into three categories: relational, task, and cognitive crafting [6]. Job crafting enhances the alignment among the cognitive, relational, and task limits, which can lead to an improved quality of care for nurses’ patients. The ability to change one’s tasks or aspects of one’s work environment is the primary trait of job crafting behaviors. This, in turn, leads to a transformation of one’s work meaning or identity, which typically results in an improvement in job performance, career competencies, and productivity [7].

The job crafting paradigm comprises four dimensions: increasing structural resources, increasing challenging job demands, growing or seeking social resources, and decreasing hindering job demands. Nurses shape their professions by modifying the number of job demands and accessible employment resources [8]. In addition, the job crafting approach makes it possible for supervisors and nurses to work together to reevaluate the purposes, responsibilities, and interactive relationship of each job, which positively influences employees in the workforce [9]. San noted three definitions of happiness - “positive thinking,” “psychological ease,” and “inner serenity,”-all of which are essential and significantly contribute to healthy living [10].

A high degree of happiness among employees is crucial, particularly in nursing wherein job tasks require providing others with care and therapy [11]. Patients require self-assured, creative, caring, and enthusiastic caretakers, and each of these attributes is directly associated with feeling happy. Nursing environments are challenging and can negatively impact employees’ mental health and capacity to deliver quality care [11]. Nurses’ happiness, an aspect of emotional well-being, comprises three major components: a degree of pleasant feelings or pleasure, the absence of negative sensations, and an average level of satisfaction at a specific period [11]. The Center for Bhutan Studies defined happiness as “a subjective state of well-being” and a component of an individual’s overall health [12].

Happiness leads to increased work performance and satisfaction, improved relationships among healthcare professionals, and higher levels of patient satisfaction. Hosseini et al. noted that nurses can possess varying degrees of happiness, largely ranging from low to moderate [13]. This may be related to the demanding nature of the occupation and the technical skills, acute awareness, and sound decision-making required for success [11].

In one study conducted in Saudi Arabia, Baghdadi et al. found that job crafting is a strong factor in nurses’ work engagement [14]. However, no researchers in Saudi Arabia have examined the relationship between job crafting and happiness among nurses. Therefore, the primary aim of the present study is to investigate the relationship between job crafting and nurses’ happiness in the Bisha region of Saudi Arabia.

According to the Kingdom’s Vision 2030, all Saudi ministries and government agencies are expected to follow best practices in human capability development, recruit individuals according to merit, and work toward developing talented individuals capable of becoming future leaders [15]. The King Salman Program for Human Capital Development will establish human resources (HR) centers of excellence in all government departments, and these centers will be responsible for providing jobs and job training to increase employee productivity by implementing appropriate performance management standards, providing continuous professional development opportunities, and sharing knowledge. These centers will develop targeted policies for identifying and empowering future leaders and foster an environment that promotes equal opportunity and rewards for excellence [15].

Significant research has demonstrated that the work attitude of nurses, frequently considered to be essential in the field of health care, is associated with the health outcomes and safety of the patients they care for. The process by which workers alter their responsibilities to discover new meaning and significance in their work and, as a result, feel a greater level of contentment in their positions is known as job crafting [16]. Job crafting is a concept that can be studied, taught, and then effectively implemented in the clinical setting. It is particularly exciting for organizations that are involved in the healthcare industry [17].

Few studies have been conducted on job crafting among healthcare personnel [18]. Similarly, to the best of the researcher’s knowledge, no research conducted in Saudi Arabia has concerned the association between job crafting and levels of happiness among nurses at work. The findings of the present study are expected to help facilitate an understanding of job crafting, its implementation within health organizations, and its potential to improve nursing care. The present findings may also narrow the information gap that has resulted from the scarcity of research on the relationship between job crafting and happiness among nurses in Saudi Arabia.

## Materials and methods

Objective and design

In this study, the researcher uses a quantitative, descriptive cross-sectional design to investigate the relationship between job crafting and nurses’ happiness. This study was conducted in the Bisha region of Saudi Arabia.

Study objective

To assess job crafting and level of happiness among nurses in the Bisha region of Saudi Arabia.

To explore the association between job crafting and happiness among nurses in the Bisha region of Saudi Arabia.

Sample and setting

The convenience sampling technique was used to recruit the study participants from each hospital in the Bisha region. A convenience sample is used because it allows the researcher to option basic data and trends regarding this study without the complication of using a randomized sample. The study was carried out at three central hospitals: 1) King Abdullah Hospital, total bed capacity of 300 beds, 2) Psychiatric and Long-Term Hospital with a bed capacity of 100 beds, and 3) Maternity and Children Hospital, total bed capacity is 100. They are nonprofit government hospitals. The current study's population consists of nurses working in the three main hospitals mentioned above. The total population was 750. All of them were subjected to being included in the process of data collection. Out of 750 nurses, 441 responded to the study questionnaire. Using the Raosoft power analysis program, the sample size was calculated. In this study, 255 participants need to be surveyed with a confidence level of 95%; a 0.5 margin of error is the minimum recommended for this study. The response rate was very high; we included all participants to increase the accuracy of the study.

The distribution of the sample from each hospital:

· King Abdullah Hospital = [443/750] x 255= 150

· Psychiatric and Long-Term Hospital = [96/750] x 255= 33

· Maternity and Children’s Hospital = [211/750] x 255= 72

Eligibility criteria and instruments

The study inclusion criteria are as follows: all nurses working in the three main aforementioned hospitals, Saudi and non-Saudi nurses, and nurses who have at least six months of experience were included to participate in the current study. An electronic questionnaire (Google Drive) was used to collect data. The questionnaire involved three parts; the first part was about the socio-demographic characteristics of participants such as age, gender, hospital name, working unit, nationality, marital status, educational level, experience, number of children, income, income status, work schedule, and working hours. The second part was the Job Crafting Scale (JCS) developed by Tims et al. [19], which included four distinct dimensions: (1) Challenging job demands (5 items); (2) Social job resources (5 items); (3) Hindering job demands (6 items); and (4) Structural job resources (5 items). Then, the participants were asked to give their responses on a five-point Likert scale from 1 (never) to 5 (Always). The scoring system was devised by a statistician into five categories based on the total subscales weighted mean range (Table 1):

**Table 1:** JCS scoring system JCS: Job Crafting Scale

Overall JCS Level	Mean Range	Weighted Mean Range
Very Low	21 to < 37.8	1 to < 1.8
Low	37.8 to < 54.6	1.8 to < 2.60
Medium	54.6 to < 71.4	2.60 to < 3.40
High	71.4 to < 88.2	3.40 to < 4.20
Very High	88.2 to 105	4.20 to 5

The third part involved the Oxford Happiness Questionnaire (OHQ) developed by Hills, Argyle, & Crossland [20]. This tool consists of 29 items, and the respondents were asked to give their responses on a six-point Likert scale ranging from 1 (strongly disagree) to 6 (strongly agree). Negative statements were reverse-coded. Higher happiness scores indicate greater contentment. This scale used a scoring as 1‐2 (not happy); 2‐3 (somewhat unhappy); 3‐4 (not particularly happy or unhappy); 4‐5 (rather happy; pretty happy); 5‐6 (very happy); and 6 (too happy).

Instrument validity and reliability

The questionnaires were translated by an expert translator into the Arabic language using a translation and back-translation technique. Face validity was checked by sending the instrument to five academic experts (three academic experts in the field of nursing administration and two academic experts from the Medical-Surgical department) in the faculty of nursing at King Abdulaziz University to test the instrument. In addition, content validity was conducted to test and assess whether the instrument can evaluate and measure all aspects of the topic.

The researchers checked the instrument’s reliability by measuring Cronbach's alpha coefficient and Guttman split-half coefficient. The total Cronbach’s alpha for job crafting was 0.902 and 0.838 for happiness; these values are considered very good.

Data collection

After obtaining ethical approval from the Bisha Directorate of Health Affairs, the researcher used Google Drive to create a link to the electronic questionnaire. Participants were recruited to participate in the research after their voluntary consent was obtained on the first page of the questionnaire. Further, the researcher ensured participant anonymity.

The researcher visited the three hospitals and spoke with the nursing director, supervisors, and head nurses at each location to explain and deliver the electronic questionnaire. Through emails and WhatsApp, the researcher distributed the questionnaire to the staff nurses. The total data collection period for the final study was from February 27, 2022, to April 3, 2022.

Data analysis

The researcher used the Statistical Package for the Social Sciences (SPSS version 26; IBM Corp., Armonk, NY) for analyzing the data. Descriptive statistics were used to describe the demographic variables of the study such as age, gender, educational level, income level, marital status, experience, etc. Frequency and percentage were used in this type of statistic. The researcher calculated the mean percentage for the total scale for job crafting and happiness by dividing the total mean score of the scale by the total number of participants and then multiplying this result by 100. Additionally, the researcher used inferential statistics to answer the research questions and a Mann-Whitney test to investigate the differences in the mean score of job crafting and work happiness regarding the demographic categorical variables (only two categories) of nationality (Saudi or non-Saudi) and gender (male or female). This test was used because of the abnormal distribution of these variables. Further, the researcher used a Kruskal-Wallis test to investigate variations in the mean score of job crafting and happiness regarding their demographic category (more than two categories) and independent variables such as hospital name, working unit, and working experience. This test was used because of the abnormal distribution of these variables.

Moreover, the researcher used a Pearson correlation test to measure the correlation between job crafting and job happiness using an R-value and a P-value was used to indicate the level of significant differences in the mean score in the Mann-Whitney and Kruskal-Wallis tests. P-values below 0.05 were considered statistically significant. Tables and figures were used to describe and present the results in detail.

Ethical considerations

In conducting this study, the researcher was committed to adhering to all ethical standards. First, the researcher obtained approval for the study from the ethical committee of the nursing department at King Abdulaziz University in Jeddah.

Second, approval needed was from the studies and research department at the Directorate of Health Affairs in Bisha; via email, this was sent to King Abdullah Hospital, the Psychiatric and Long-Term Hospital, and the Maternity and Children’s Hospital to facilitate the researcher’s mission. Finally, the researcher received permission from Dr.Maria Tims to use his Job Crafting Scale and to translate the material into Arabic.

Before starting the data collection, the researcher received informed consent from all respondents. The study was conducted anonymously - this method was explained before the completion of consent forms. Further, the participants assumed no related risks by participating in this study. Upon completion, the answered questionnaires were stored with the researcher in a secure location to maintain data confidentiality; the responses will be deleted once the study has been completed.

In addition, participants were not obligated to participate in the study and were free to decide if they wanted to participate. Further, the participants could withdraw from participation at any point.

## Results

Demographic and professional characteristics

A total of 441 nurses responded to the survey. Table 2 presents the nurses’ sociodemographic and professional characteristics. The majority of the nurses (41.5%) were in the age group of 30-40, and nearly all (95.7%) were female. Approximately 57.6% of the nurses were working in King Abdullah Hospital in mostly ICU or ER units (31.5%). Non-Saudi nurses constituted 67.8%, and 56% of nurses were married. Regarding education, most participants had at least bachelor’s degrees (81%), and 40.6% had less than five years of experience in the field. More than one-third of participants (34.7%) had one to two children. The results also demonstrate that although 55.1% had an income of 5,000-10,000 SAR per month, 69.6% stated that their income status was insufficient. Further, nurses working in rotation comprised 57.1% of the total, and nurses working for 12 hours comprised 66% of the total.

**Table 2:** The distribution of nurses according to the sociodemographic and professional characteristics of nurses (n=441)

Demographic Characteristics	N	%
Age Group		
< 30 years	170	38.5%
30–40 years	183	41.5%
> 40 years	88	20.0%
Gender		
Male	19	04.3%
Female	422	95.7%
Hospital		
King Abdullah Hospital	254	57.6%
Maternity and Children’s Hospital	142	32.2%
Psychiatric and Long-Term Care Hospital	45	10.2%
Working Unit		
Intensive care unit (ICU)/emergency room (ER)	139	31.5%
Operating rooms	41	09.3%
Outpatient department	38	08.6%
General ward	104	23.6%
Other	119	27.0%
Nationality		
Saudi	142	32.2%
Non-Saudi	299	67.8%
Marital Status		
Unmarried	167	37.9%
Married	247	56.0%
Education Level		
Diploma	78	17.7%
Bachelor’s degree	357	81.0%
Master’s degree or Ph.D.	06	01.4%
Working Experience (Years)		
< 5	188	42.6%
5–10	115	26.1%
> 10	138	31.3%
Number of Children		
0	224	50.8%
1–2	153	34.7%
3–4	52	11.8%
> 5	12	02.7%
Monthly Income (SAR)		
< 5,000	125	28.3%
5,000–10,000	243	55.1%
> 10,000	73	16.6%
Income Status		
Enough	134	30.4%
Not enough	307	69.6%
Work Schedule		
Fixed	189	42.9%
Rotating	252	57.1%
Working Hours		
6	04	0.90%
8	146	33.1%
12	291	66.0%
Total	441	100%

Descriptive statistics for Job Crafting Scale domains

Table 3 displays the descriptive statistics for JCS and its domain. The mean score was highest in decreasing hindering job demands domain (26.5 ± 3.68) and lowest in the increasing social job resources domain (20.2 ± 4.49). The total mean score of JCS was 91.2 (SD 11.8), which indicates that nurses had a very high level of job crafting.

**Table 3:** Mean and standard deviation for job crafting domains (n = 441) JCS: Job Crafting Scale; SD: standard deviation *Responses recorded via a five-point Likert scale from 1 (“never”) to 5 (“always”).

JCS Domains	Mean	SD	Rank
Increasing structural job resources	23.5	2.31	2
Decreasing hindering job demands	26.5	3.68	1
Increasing social job resources	20.2	4.49	4
Increasing challenging job demands	21.0	3.85	3
Total JCS Score	91.2	11.8	--

Classification of happiness

**Figure 1:**
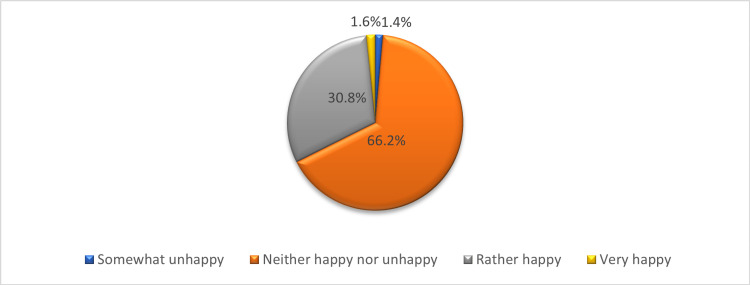
Distribution of nurses according to the level of happiness

In Figure 1, 66.2% of the nurses were classified as neither happy nor unhappy, 30.8% were rather happy, and only 1.4% were very happy in their workplace environment.

Correlation between OHQ scores and JCS scores and their domains

In Table 4, the researcher observed a positive significant correlation among OHQ scores according to the increasing structural domain (r = 0.246), decreasing hindering job demands (r = 0.220), increasing social job resources (r = 0.176), increasing challenging job demands (r = 0.212), and overall total JCS (r = 0.252). This indicates that an increase in job happiness is correlated with an increase in job crafting (Table 3).

**Table 4:** Correlation (Pearson-r) between OHQ scores and JCS scores and their domains (n = 441) JCS: Job Crafting Scale; OHQ: Oxford Happiness Questionnaire **Correlation is significant at the 0.01 level (2-tailed).

JCS Domains	OHQ Score	
	R-value	P-value
Increasing structural job resources	0.246	< 0.001**
Decreasing hindering job demands	0.220	< 0.001**
Increasing social job resources	0.176	< 0.001**
Increasing challenging job demands	0.212	< 0.001**
Total JCS	0.252	< 0.001**

Differences in the scores of JCS and OHQ in relation to the sociodemographic of nurses 

In Table 5, it was found that a higher JCS score was more in nurses who worked in psychiatric and long-term care hospitals (H=10.600; p=0.005), working in the outpatient unit (H=13.537; p=0.009), being married (Z=2.513; p=0.012), being a diploma holder (Z=2.707; p=0.007), fixed working schedule (Z=2.035; p=0.042) and six to eight working hours per day (Z=3.052; p=0.002) while the differences in the score of JCS among the age group, nationality, work experience, having children, and income status did not reach statistical significance (p>0.05).

**Table 5:** Differences in the scores of JCS and OHQ in relation to the sociodemographics of nurses (n=441) a P-value has been calculated using the Kruskal-Wallis H-test. b P-value has been calculated using the Mann-Whitney Z-test. ** Significant at the p<0.05 level.

Factor	JCS Score (105) Mean ± SD	H/Z-test; P-value	OHQ Score (6) Mean ± SD	H/Z-test; P-value
Age group ^a^				
<30 years	90.6 ± 12.7	H=0.948; P=0.623	3.82 ± 0.42	H=11.635; P=0.003 **
30 – 40 years	91.9 ± 11.4		3.91 ± 0.45	
>40 years	90.9 ± 10.9		3.97 ± 0.38	
Hospital name ^a^				
King Abdullah Hospital	91.6 ± 11.5	H=10.600; P=0.005 **	3.91 ± 0.44	H=4.284; P=0.117
Maternity and Child Hospital	89.2 ± 12.6		3.85 ± 0.41	
Psychiatric and long-term care Hospital	95.5 ± 10.0		3.85 ± 0.38	
Working unit ^a^				
ICU/ER	88.8 ± 12.6	H=13.537; P=0.009 **	3.79 ± 0.38	H=18.496; P=0.001 **
Operating rooms	90.9 ± 13.2		3.90 ± 0.55	
Outpatients	96.1 ± 10.3		3.95 ± 0.43	
General wards	91.8 ± 11.2		3.87 ± 0.40	
Other unit	92.2 ± 10.8		3.97 ± 0.43	
Nationality ^b^				
Saudi	92.5 ± 10.5	Z=1.305; P=0.192	3.97 ± 0.48	Z=2.538; P=0.011 **
Non-Saudi	90.6 ± 12.4		3.85 ± 0.39	
Marital status ^b^				
Unmarried	89.4 ± 12.9	Z=2.513; P=0.012 **	3.89 ± 0.46	Z=0.530; P=0.596
Married	92.7 ± 10.7		3.88 ± 0.39	
Educational level ^b^				
Diploma	94.5 ± 10.3	Z=2.707; P=0.007 **	3.95 ± 0.38	Z=2.161; P=0.031 **
Bachelor or higher	90.5 ± 12.0		3.87 ± 0.43	
Working experience ^a^				
<5 years	91.7 ± 11.8	H=1.624; P=0.444	3.83 ± 0.42	H=9.726; P=0.008 **
5 – 10 years	89.9 ± 12.8		3.88 ± 0.45	
>10 years	91.7 ± 89.9		3.96 ± 0.39	
Having children ^b^				
Yes	92.5 ± 10.6	Z=1.691; P=0.091	3.89 ± 0.39	Z=0.530; P=0.596
No	89.9 ± 12.8		3.88 ± 0.45	
Income status ^b^				
Enough	90.9 ± 11.8	Z=0.584; P=0.559	4.01 ± 0.45	Z=4.023; P<0.001 **
Not enough	91.4 ± 11.9		3.83 ± 0.40	
Work schedule ^b^				
Fixed	92.5 ± 11.7	Z=2.035; P=0.042 **	3.96 ± 0.44	Z=3.123; P=0.002 **
Rotating	90.3 ± 11.8		3.83 ± 0.40	
Working hours ^b^				
Six to eight hours	93.8 ± 10.2	Z=3.052; P=0.002 **	3.98 ± 0.43	Z=3.442; P=0.001 **
Twelve hours	89.9 ± 12.4		3.84 ± 0.42	

A higher OHQ score was more in nurses being of older age (H=11.635; p=0.003), being of Saudi nationality (Z=2.538; p=0.011), being a diploma holder (Z=2.161; p=0.031), having work experience of more than 10 years (H=9.726; p=0.008), having enough monthly income (Z=4.023; p<0.001), having fixed working schedules (Z=3.123; p=0.002), and working for six to eight hours a day (Z=3.442; p=0.001). Regarding working units, a higher score was for nurses working in other units while a lower OHQ score was more for nurses working in the ICU/ER unit (H=18.496; p=0.001). Other variables, such as hospital name, marital status, and having children, were not statistically significant when compared to the OHQ score (p>0.05).

## Discussion

Nurses are crucial factors in the efficiency, efficacy, and sustainability of healthcare systems. Therefore, it is crucial to determine the factors that drive hospital employees to remain in their positions. While poor working conditions and organizational cultures are significant determinants of nurses’ employment discontentment, job crafting behaviors are proactive behavioral treatments whereby nurses reshape, redesign, or adapt their occupations to improve their health, motivation, and job satisfaction [5]. In the present study, the researcher investigated the association between job crafting and nurse happiness in Saudi Arabia.

The results revealed that most nurses had a very high level of job crafting; this may be the result of nurses being aware of the idea of job crafting and the ways in which it can be used in the clinical setting. In addition, the work environment may present nurses with opportunities to grow their professional talents or actively engage in the decision-making process, both of which have positive outcomes. The findings of the present study suggest that managers may provide feedback to nurses on activities related to job crafting or coaching nurses to independently remodel their employment.

The current results are consistent with those of Baghdadi et al., who demonstrated that engaged nurses had high levels of job crafting [14]. The present results, however, differ from those of Huang et al., who demonstrated that the mean score of overall job crafting was at a moderate level [21]. Moreover, decreasing hindering job demands had the highest percentage score; this finding aligns with that of Cheng et al., who determined that the highest score was achieved for the reduction of job demands that were a hindrance to job performance [22]. In addition, the present findings contradict the findings of Badran and Akeel, who reported that the structural job resources component of job crafting had the highest mean score [23]; this difference may be attributed to the setting of the current study, which entailed significant workloads and highly stressful working conditions. Rudolph et al. noted that nurses who experience higher workloads will be motivated to reduce the demands that create obstacles and seek resources that can help them counteract those demands [24]. As a result, decreasing job demands may have the unintended consequence of workloads being piled onto coworkers [25].

In contrast, increasing social work resources received the lowest possible score; this may be attributed to inadequate frameworks of social support that may not enhance individuals’ workplace opportunities. The factor that received the lowest mean score was the one that had the most negative impact on work demands. Because of the apparent vitality of the social elements of job crafting, recent studies have underlined the need for additional research on this social perspective, e.g., Zhang & Parker and Rofcanin Y [6,26]. Social support at work had a significant favorable effect on the development of social job resources [27].

Regarding happiness among nurses, the present results demonstrate that the overall mean happiness score was a moderate level of happiness; this result is similar to that of Kose et al., who found that the mean scores of happiness for nurses who had an average working time of 120-180 hours per month were at moderate levels [28]. The present result is similar to what was revealed by Javadi Sharif et al., who noted that the mean score of happiness was 55.3%, suggesting an average level of happiness among nurses [11]. Like our findings, one study conducted in China showed moderate levels of happiness among nurses [29]. Overall, the current study’s findings are similar to those that exhibited a moderate degree of happiness among hospital nurses [30]. Most studies indicate that hospital nurses experience a low to average degree of happiness, which may be related to the negative emotions they experience when caring for patients as well as their difficult working conditions and high workload [30].

The top five highest mean ratings belonged to the following statements: “Life is good,” “I am always committed and involved,” “I feel fully mentally alert,” “I have very warm feelings toward almost everyone,” and “I find beauty in some things,” while the lowest rating was related to the statement “I don’t find it easy to make decisions.” This finding indicates that nurses express feelings of optimism but have some difficulties in making decisions as a result of working in a stressful environment. Nurses frequently make professional decisions in healthcare situations while serving the needs of patients; they make decisions based on their knowledge and expertise as part of their positions in administration, research, education, and healthcare practices [31]. Similarly, as noted by Nibbelink and Brewer, nurses make critical decisions about patient treatment, organizational events, and professional situations [32]. They are under pressure to adapt to increasingly demanding and complex healthcare environments and the clinical care settings in which they work.

The present results reveal a significant positive correlation among OHQ scores related to the increasing structural domain, decreasing hindering job demands, increasing social job resources, increasing challenging job demands, and overall total JCS. This indicates that the increase in nurse happiness is correlated with the intensification in job crafting level. The result of this study is not comparable to the findings of Abou Shaheen and Aly Mahmoud, which demonstrated the majority of nurses had a low level of job crafting due to a lack of understanding of the job. It also found the majority of nurses had a low level of satisfaction [33]. These results may be due to the nurses working in university hospitals being under a harsh atmosphere and usually being overburdened with an increased number of patients and, simultaneously, without a financial reward appropriate to their effort. In addition, there was a negative significant correlation between job crafting and counterproductive work behavior. This means that the nurses who craft their job are less likely to engage in counterproductive work behavior.

Further, the findings of this study are comparable to those of Chang et al. in Korea, who found that nurses who are happy with their jobs are considerably more likely to engage in job crafting, which positively influences both the activities of workers, such as their desire to create meaning and worth in their job and the organization they work for. Further, Chang et al. demonstrated that job crafting results in positive effects such as increased organizational commitment, increased job satisfaction, improved psychological health, and successful productivity outcomes [16].

The findings shown here are corroborated by those of Polatci and Sobaci, who demonstrated that job crafting has a causal effect on work satisfaction [34]. In addition, Naami determined that there were significant connections among subscales of job crafting and work engagement, job satisfaction, and emotional commitment [35], again consistent with the findings of the present study.

The current study results are in line with the results of previous studies, demonstrating that nurses were more likely to exhibit higher levels of job crafting; the present findings indicate that nurses who reported greater job crafting were more likely to be happy. In addition, Seligman and Csikszentmihalyi found that those content with their lives were more likely to be self-motivated in their efforts to redesign their work [36]; in this scenario, happy nurses would have higher levels of self-esteem, quality job performance, and passion for their professional roles. Because all these factors are associated with job crafting, happy nurses would exhibit proactive attitudes toward crafting their jobs and roles at work.

The significant correlation between job happiness and job crafting may be explained by the fact that both structural job resources and increasing social job resources contribute to an increase in participants’ levels of happiness. Similarly, Baghdadi et al. demonstrated that job crafting is a crucial element in influencing the amount of work engagement that nurses experience in their jobs [14].

Moreover, Sidin et al. showed in a study in Mamuju that job crafting can contribute to employee happiness at work when other supportive elements are also present [37]. In addition, the findings presented here are in line with those presented by Yepes-Baldó et al. in Spain and Sweden [38]; the authors demonstrated a positive linear association among job crafting, well-being, and quality of care in Spain.

The current study results revealed that a higher JCS score was more associated with working in psychiatric and long-term care hospitals, working in the outpatient unit, being married, being less educated, having a fixed working schedule, and working six to eight hours per day while the differences in the score of JCS among the age group, nationality, work experience, having children, and income status did not reach statistical significance.

The most recent findings demonstrated a statistically significant correlation between the number of years of nursing experience a nurse had and their overall work crafting behaviors. This outcome is comparable to what Shusha has disclosed in his research [39]. The current outcome may be understood if one considers the fact that experienced nurses are making full use of their capabilities to shape their work. Also, diploma nurses had more experience, which is beneficial to nurses in their pursuit of job crafting.

Demographic factors associated with higher happiness among nurses included older age, Saudi nationality, having a diploma, having more than 10 years of work experience, having a sufficient monthly income, having a fixed working schedule, and working for six to eight hours a day. A lower happiness score was more associated with working in ICU or ER units. In contrast, marital status, having or not having children, and the hospital name did not affect the level of nurses’ happiness in the present study.

Regarding the working department (ICU/ER), the current result differs from that of Jun et al., who demonstrated no correlation between the type of ward and the mean happiness score of nurses in Korea [40]. In contrast, the present study findings are consistent with the results of Khosrojerdi et al., who demonstrated that happiness scores were significantly affected by the type of unit nurses work in [30].

In this study, a lower score of happiness was observed among nurses working in either the ICU or ER unit; this may be attributed to the heavy work demand and workload experienced by nurses in those departments. In the ICU, nurses experienced physical lifting, long periods of standing, intensive caring, and significant levels of stress. Moreover, ER nurses experience sudden traumas, a large number of patients, and different types of patients, with different needs for each patient. All these factors can affect the level of happiness score of nurses working in these departments.

Regarding education level, the current result is not like what was revealed by Javadi Sharif et al. [11], who noted no significant differences in the mean score of happiness with regard to nurses’ education levels. The current result may be attributed to the type of work experienced by diploma nurses compared to nurses with bachelor’s degrees. Diploma nurses have fewer work demands and fewer responsibilities compared to those with bachelor’s degrees; diploma nurses in the Ministry of Health may work less frequently in critical care units. Additionally, diploma nurses had more life experience and may have become more familiar with various life and work scenarios, which, in turn, affected their happiness scores.

Regarding the level of income, the current result is not similar to that of Javadi Sharif et al., who demonstrated no significant differences in the mean score of happiness with regard to nurses’ level of income [11]. In contrast, similar results were found by Khosrojerdi et al., who showed that nurses’ income was associated with the level of happiness among nurses [30]. In the present study, the impact of income on happiness among nurses may be attributed to the fact that with an increase in salary, the psychological status of an individual would be enhanced. This will help nurses improve their lives and live in good conditions, which, in turn, will affect their happiness scores.

Regarding the fixed schedule, the current result is not similar to what was revealed by Javadi Sharif et al., who showed no significant differences in the mean score of happiness with regard to the fixed schedule [11]. In contrast, similar results were found by Khosrojerdi et al., demonstrating a significant difference in the mean score of happiness regarding fixed schedules [30]. Fixed schedules decrease life stress experiences for nurses because they make their life situations and events more organized, and they organize their lives through daily routine and social issues based on their schedule in the hospital.

Regarding nurses’ age, the current result is not similar to that of Javadi Sharif et al. [11], who showed no significant differences in the mean score of happiness with regard to nurses’ age. By contrast, similar results were found by Khosrojerdi et al. [30], who revealed a significant difference in the mean score of happiness regarding nurses’ age. The present study findings may be attributed to the fact that, with an increase in age, individuals gain more experience and become more familiar with dealing with life and work situations and stress.

Regarding nurses’ years of experience, the current result is not similar to what was revealed by Javadi Sharif et al., who showed no significant differences in the mean score of happiness with regard to nurses’ years of experience [11]. In the present study, having more experience is associated with a higher happiness score, and this may be attributed to the experience gained from several years of work experience.

Regarding the length of working hours, the current result is similar to that of Khosrojerdi et al., who revealed a significant difference in the mean score of happiness with regard to the length of working hours [30]. In the present study, with an increase in working hours, the level of happiness decreased owing to an increase in job demand and workload as well as heavy mental stress.

Limitations of the study

Interpreting the study’s findings about the relationship between job crafting and nurses' happiness requires the consideration of some imitations. First, some units in King Abdullah Hospital had patients infected with COVID-19, and thus the researcher was not allowed to enter to distribute the questionnaire or contact individuals. In this case, the researcher was asked to send the survey through an email or WhatsApp, which was not congruent with the researcher’s data collection process.

Second, the use of a single research city can be a hindrance to the effective representation of the studied phenomenon. In the present study, the researcher relied on a single research city owing to transportation and financial restraints, thus limiting the generalizability of the study’s findings.

Nursing administration recommendations

Providing options for increasing social job resources is important for both nurses and hospitals because it leads to nurses’ retention and recruitment through training programs that focus on empowering nurses with the tools necessary to ask for feedback or coaching while maintaining standard clinical protocols.

Managers should provide nurses with adequate authority to determine how to improve their work environment related to enhancing the accessibility of job resources and minimizing hindering job demands to develop career capabilities. For example, managers should educate staff members on job-crafting strategies and encourage employees to take the initiative when they want more challenging work or less hindering job requirements.

Nursing education recommendations

In-service education and training can be used to organize special lectures and training programs for nurses related to job crafting and encourage nurses to be happy during their work. Educational departments should provide job-crafting training programs for nurses so that they are better equipped to handle employment challenges.

Research recommendations

Future job-crafting intervention studies should be conducted. Also, future researchers should examine the long-term effects of job crafting on happiness and well-being among nurses Likewise, it is possible that other variables, such as workload, support from coworkers and supervisors, and overall job satisfaction, may also influence the relationship between job crafting and happiness. Thus, authors of additional studies in this field should consider the potential role of these confounding variables to better understand the relationship between job crafting and happiness.

## Conclusions

Job crafters are more satisfied at work, give efficient nursing care, and assist hospitals in achieving organizational objectives. According to our research, happiness at the personal level, instead of nursing practice settings at the executive level, was strongly connected with job crafting among nurses. Administration support, like leisure activity programs and low overtime, should promote nurses' contentment and work crafting. Institutions should also identify the facilitators and obstacles to nurses' work crafting across jobs and unit types and offer various educational and training facilities to encourage job crafting. These organizational initiatives may help boost employee happiness and create an atmosphere where nurses may be happy, mainly if they must stay at work for long periods. To acquire representative results, future research should incorporate bigger sample sizes from numerous places. Furthermore, further research should identify evidence-based techniques to enhance nurse happiness and job crafting. Finally, it is important to test what increases the job-crafting attitude.
